# Supplementary material for: Association of Non-Alcoholic Fatty Liver Disease and Hepatic Fibrosis with Epicardial Adipose Tissue Volume and Atrial Deformation Mechanics in a Large Asian Population Free from Clinical Heart Failure
Source: Diagnostics (Basel). 2022 Apr 6;12(4):916. doi: 10.3390/diagnostics12040916 (PMC9033151; doi:10.3390/diagnostics12040916)
Supplement: Supplementary file 1 [file diagnostics-12-00916-s001.zip › diagnostics-1655405-supplementary.pdf]

Supplementary Table S1: Baseline demographics, adiposity measures and biomarkers after excluding the youngest quartile from non-fatty liver group

	Non-fatty liver	NAFLD, low fibrosis score (<-1.455)	NAFLD, high fibrosis score (≥-1.455)	P_{trend}
	**N=764**	**N=840**	**N=302**	
Age, years	50.64(9.1)	47.69(9.1)*	56.37(8.34)*†	<0.001
Female sex, %	390 (51.0%)	211 (25.1%)*	76 (25.2%)*	<0.001
SBP, mmHg	118.48(15.28)	123.86(16.42)*	129.21(16.03)*†	<0.001
DBP, mmHg	73.99(9.93)	78.04(10.21)*	80.45(10.06)*†	<0.001
Adiposity measures				
EFV, ml	63.65(25.67)	79.73(26.25)*	95.13(31.67)*†	<0.001
BMI, kg/m2	22.33(2.49)	25.69(3.17)*	27.21(3.53)*†	<0.001
WC, cm	77.95(7.75)	87.29(8.24)*	91.06(9.46)*†	<0.001
Body fat, %	23.96(6.45)	27.42(7.38)*	28.9(7.61)*†	<0.001
Biomarkers				
Fasting glucose, mg/dl	94.01(10.15)	100.15(18.17)*	118.17(32.1)*†	<0.001
HbA1c, %	5.59(0.42)	5.74(0.66)*	6.32(1.18)*†	<0.001

Fasting insulin, U/L	6.53(3.71)	10.01(6.16)*	10.86(5.81)*	<0.001
HOMA-IR	1.51(0.92)	2.5(1.78)*	3.23(2.05)*†	<0.001
Hs-CRP, mg/L	1.56(4.63)	2.42(4.16)*	2.67(3.73)*	0.001
GOT, IU/L	21.56(6.38)	26.11(11.3)*	28.1(14.57)*†	<0.001
GPT, IU/L	21.59(10.65)	36.42(24.24)*	34.44(21.94)*	<0.001
TC, mg/dL	204.76(35.84)	211.29(36.5)*	207.02(35.7)	<0.001
TG, mg/dL	106.81(58.96)	167.19(95.16)*	170.79(111.15)*	<0.001
LDL-C, mg/dL	130.61(33.9)	140.58(33.38)*	136.89(31.84)*	<0.001
HDL-C, mg/dL	61.28(16.04)	48.77(12.45)*	47.96(11.41)*	<0.001
eGFR, ml/min/m2	89.69(15.93)	89.3(15.2)	85.39(16.84)*†	<0.001

Data presented as mean(SD)

P-value<0.05 for comparisons against * Non-fatty liver, † Fatty liver with low fibrosis score

Abbreviations: SBP=systolic blood pressure, DBP=diastolic blood pressure, EFV=epicardial fat volume, BMI=body mass index, WC=waist circumference, HOMA-IR=homeostasis model assessment-insulin resistance, Hs-CRP=high-sensitivity C-reactive protein, GOT=glutamic oxaloacetic transaminase, GPT=glutamate pyruvate transaminase, TC=total cholesterol, TG=triglyceride, LDL-C=low-density lipoprotein cholesterol, HDL-C=high-density lipoprotein cholesterol, eGFR=estimated glomerular filtration rate

Supplementary Table S2: Echocardiographic parameters after excluding the youngest quartile from non-fatty liver group

	Non-fatty liver	NAFLD, low fibrosis score (<-1.455)	NAFLD, high fibrosis score (≥-1.455)	P_{trend}
	N=1019	N=840	N=302	
LVST, mm	8.69(1.02)	9.14(0.96)*	9.54(1.02)*†	<0.001
LVPT, mm	8.69(0.92)	9.13(0.88)*	9.48(0.95)*†	<0.001
RWT	0.38(0.04)	0.39(0.04)*	0.4(0.04)*†	<0.001
LVEDV, ml	72.51(13.36)	77.26(12.48)*	80.75(11.04)*†	<0.001
LVEF, %	62.92(5.02)	62.16(5.2)*	62.34(5.08)	0.01
LVM, gm	131.35(30.11)	146.4(27.82)*	159.65(30.33)*†	<0.001
LVMi(BSA), gm/m^2	73.84(13.99)	74.89(12.56)	80.42(13.79)*†	<0.001
LVMi, $gm/m^{2.7}$	34.66(7.22)	36.86(7.11)*	41.08(7.94)*†	<0.001
LAV, ml	26.94(9.0)	31.44(11.22)*	35.63(12.48)*†	<0.001
LAEF, %	58.61(10.46)	57.72(10.73)	56.94(10.48)	0.05
Diastolic function				
DT, ms	199.28(39.02)	201.19(35.32)	214.5(40.65)*†	0.001

IVRT, ms	88.98(14.14)	89.87(13.64)	94.03(18.93)*†	<0.001
E/A	1.29(0.43)	1.19(0.37)*	1.04(0.36)*†	<0.001
LV e', cm/sec	9.62(2.09)	9.11(2.04)*	7.85(1.88)*†	<0.001
LV s', cm/sec	8.3(1.45)	8.32(2.07)	7.98(1.45)*†	0.01
E/e'	7.03(2.28)	7.11(2.43)	8.08(3.05)*†	<0.001
Strain indices				
LV GLS, %	20.75(1.93)	19.78(1.6)*	19.53(1.71)*	<0.001
PALS, %	39.38(7.44)	36.96(7.77)*	34.05(8.01)*†	<0.001
$ALSR_{syst}$	1.76(0.39)	1.66(0.36)*	1.52(0.35)*†	<0.001
$ALSR_{early}$	1.88(0.5)	1.66(0.49)*	1.35(0.45)*†	<0.001
$ALSR_{late}$	2.04(0.49)	2.07(0.5)	2.05(0.49)	0.55
LA_{stiff}	0.18(0.08)	0.2(0.09)*	0.26(0.15)*†	<0.001

Data presented as mean(SD)

P-value<0.05 for comparisons against * Non-fatty liver, † Fatty liver with low fibrosis score

Abbreviations: LVST=left ventricular septal wall thickness, LVPT=left ventricular posterior wall thickness, RWT=relative wall thickness, LVEDV=left ventricular end-diastolic volume, LVM=left ventricular mass, LVMi=left ventricular mass index, LAV=left atrial volume, LAEF=left atrial emptying fraction, DT=deceleration time, IVRT=isovolumetric relaxation time, E/A=early-to-late diastolic mitral inflow velocity ratio, e'=early-diastolic tissue Doppler velocity, s'=systolic tissue Doppler velocity, GLS= global longitudinal strain, PALS=peak atrial longitudinal strain, ALSRsyst=atrial longitudinal strain rate-systolic phase, ALSRearly=atrial longitudinal strain rate-early diastolic phase, ALSRlate=atrial longitudinal strain rate-late diastolic phase, LAstiff=LA stiffness
